# Trust Games and Beyond

**DOI:** 10.3389/fnins.2019.00887

**Published:** 2019-09-10

**Authors:** Carlos Alós-Ferrer, Federica Farolfi

**Affiliations:** Department of Economics, Zurich Center for Neuroeconomics, University of Zurich, Zurich, Switzerland

**Keywords:** trust, trustworthiness, reciprocity, survey measures, social preferences, oxytocin, theory of mind, social neuroscience

## Abstract

Trust is fundamental for the stability of human society. A large part of the experimental literature relies on the Trust Game as the workhorse to measure individual differences in trust and trustworthiness. In this review we highlight the difficulties and limitations of this popular paradigm, as well as the relations to alternative instruments ranging from survey measures to neurochemical manipulations and neuroimaging.

## 1. Introduction

Trust is an essential ingredient of economic life. We implicitly or explicitly trust our financial institutions, employers, coworkers, and fellow citizens on a daily basis. Without trust, nobody would accept intrinsically-valueless bills and coins (or electronic transfers) in exchange for goods or services, or show up to work in exchange for the promise of later compensation. Yet, in spite of its fundamental importance, trust is an elusive concept which remains hard to quantify. How can we measure heterogeneity in trust or trustworthiness? Is there a quantifiable, measurable way to show that certain institutions foster (increase) trust?

Experimental economics has developed a small family of stylized paradigms used for precisely this purpose. They build on a bare-bones conceptualization of trust, which, in our view, is as follows. Trust is revealed when an agent performs an initial *sacrifice*, that is, an action which, depending on the reaction of another agent, might be detrimental to the first agent's own interests. *You put yourself in somebody else's hands*. Trust is repaid, and the second agent is revealed to be trustworthy, if his or her reaction offsets and compensates the first agent's sacrifice. Obviously, for such a situation to reflect trust and trustworthiness, the interaction must be isolated and free of any extraneous elements as might arise from strategic concerns due to repetition, coercion, etc. For this purpose, experimental economics has relied on paradigms which can be seen as *games* in the stringent sense of *game theory* (von Neumann and Morgenstern, 1944): complete descriptions of interpersonal, strategic problems. Since the very idea of trust requires a temporal structure, one ends with an *extensive form game* (e.g., Alós-Ferrer and Ritzberger, 2016), where some agent can observe and react to previous actions of another agent, creating the need for the latter to predict and forecast the reactions of the former.

The essence of the *Trust Game*, extensively used in economics as an experimental, incentivized measure of trust, is as follows. A first agent, called the *trustor*, is given a monetary endowment *X*, and can choose which fraction *p* of it (zero being an option) will be sent to the second agent, called the *trustee*. The transfer *p*·*X* is then gone, and there is nothing the trustor can do to ensure a return of any kind. Before the transfer arrives into the trustee's hands, the transfer is magnified by a factor *K* > 1 (e.g., doubled or tripled). That is, the trustor might send, say, $5 but the trustee receives $10 or more. The trustee is free to keep the whole amount without repercussion. Crucially, however, the trustee has the option to send a fraction *q* of the received transfer back to the trustor, hence honoring the trustor's initial sacrifice. Since *p* and *q* can in principle be any proportion, this is an infinite game, although in practice experimental implementations discretize the decisions, for instance requiring transfers to be integers. In the laboratory, roles are assigned randomly, the trustor-trustee matching is equally random, and interactions are computerized, one-shot, and anonymous, with the aim of isolating the essence of trust and trustworthiness.

**Figure 1:**
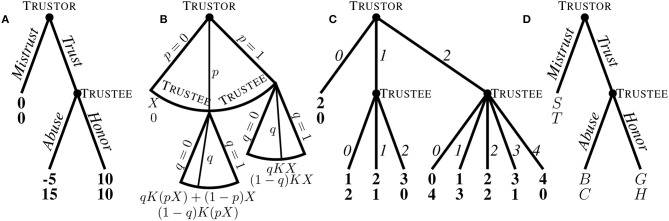
**(A)** The original Trust Game of Kreps (1990), where both trustor and trustee have just binary choices. **(B)** A qualitative depiction of the infinite game representing the continuous version of the Trust Game of Berg et al. (1995), as envisioned in experimental economics. The trustor is endowed with $X and can send any proportion *p* ∈ [0, 1]. The transfer *pX* is multiplied by a factor *K* > 1. The trustee receives *K*(*pX*) and can send back any proportion thereof, *q* ∈ [0, 1]. The trustee has a continuum of possible, alternative decision nodes, corresponding to all possible transfers by the trustor. **(C)** A discretized version of the Trust Game. The trustor is endowed with *X* = 2 and transfers are doubled (*K* = 2). Both trustor and trustee can only send integer amounts. **(D)** A heavily-discretized “mini-Trust Game,” as used, e.g., in Bohnet and Zeckhauser (2004). In all games, the upper payoff is the trustor's and the lower payoff is the trustee's.

The game described above is universally referred to as the *Trust Game* nowadays, and it is in this sense that we will use this name. However, the game was originally called the *Investment Game* by Berg et al. (1995), who used an endowment of *X* = *$*10 and tripled the transfer, *K* = 3. Further, the name *Trust Game* was used for an earlier and simpler game by Kreps (1990). In that version, the trustor has the binary choice to trust the trustee or not, with payoffs of $0 for both players if no trust is shown. In case the trustor decides to trust, the trustee faces a binary choice to either honor it, leading to equal payoffs of $10 for each player, or abuse the demonstrated trust, resulting in a payoff of $15 for the untrustworthy trustee and a negative payoff of −*$*5 for the unhappy trustor. Figure 1 presents standard game-theoretic depictions of Kreps's (1990) game (a), Berg et al.'s (1995) continuous game (b), an example of a discretized version where only integer transfers are allowed (c), and, for later reference, a mini-Trust Game with binary choices and general payoffs (d). In the latter, the structure of the Trust Game is preserved if *G* > *S* > *B* and *C* > *H* > *T*, since in this case the ordinal preferences among outcomes is preserved: The trustor would prefer to trust if trust is repaid, but not if trust is abused, the trustee's payoffs are maximized by betraying trust, but there is an option where trust is repaid and both players are better off than if no trust is shown. A binary version of the game of Berg et al. (1995), where the amount transferred by the trustor is multiplied by *K* > 1 and the trustee decides on a split of the transfer, obtains if, additionally, *B* + *C* = *G* + *H* = *K* · *S*.

While the structure of Kreps's (1990) game is different (and does not correspond to a Trust Game as currently understood), both games share four crucial features, which were put forward by Coleman (1990) to define a trust situation. First, the trustor's decision to trust is voluntary. Second, there is a time lag between the trustor's and the trustee's choices. Third, the possibility for the trustee to abuse or honor the demonstrated trust occurs if and only if the trustor does indeed show trust. And last, in case the trustee decides to (fully) abuse the demonstrated trust, the trustor will be left worse off than if no trust had been shown; that is, the trustor becomes vulnerable by exercising trust (Fehr, 2009). We would like, however, to add a fifth element to the list for a trust situation to become economically interesting: from the point of view of economic efficiency, trust should be optimal, at least in the sense of maximizing the sum of payoffs [in Kreps's (1990) version, efficiency further requires that trust is repaid; this is not the case in Berg et al.'s (1995) game, as seen most clearly comparing (a) and (d) in Figure 1 for the multiplier case, *B* + *C* = *G* + *H* = *K* · *S*].

As a consequence of these elements, rational but selfish agents fare poorly in these and similar games. A selfish trustee will never send any money back (*q* = 0), and, anticipating this (as built into the game-theoretic solution of *subgame-perfect equilibrium*), a selfish trustor will never make any transfer (*p* = 0). Needless to say, actual human beings are far more trusting and more trustworthy in the laboratory than selfishness would imply. The actual transfers of trustors can then be construed as a measure of trust, and the reactions of the trustees as a measure of trustworthiness [both becoming continuous measures in Berg et al.'s (1995) version].

The Trust Game was put forward at a time when the experimental literature was developing paradigms to measure not only trust, but also many other related constructs such as fairness or reciprocity. As a result, cross-fertilization or simply convergence of ideas is often apparent in experimental designs in behavioral economics. For example, the *Dishonest Salesman Game* (Dasgupta, 1988) framed the interaction as the purchase of a car at price β ∈ (0, 1), where the salesman can hand over a reliable car (for a utility of 1 for the buyer and α > 0 to himself) or a lemon (for a utility of zero for the buyer and γ > α for himself). A transformation of payoffs shows that this game is ordinally identical to Kreps's (1990). In the *Trading Game* of Lyons and Mehta (1997), after a previous, non-binding agreement, a Supplier decides how much to invest (say, effort or capital) and then a Buyer decides whether to pay as agreed or delay (unilaterally renegotiate the terms down). Other prominent examples have embedded trust-based interactions in more complex paradigms. For instance, in the basic building block of the *Gift-Exchange Game* (Fehr et al., 1993), employers make wage offers which employees can repay with appropriate effort levels. Employees have no incentive to provide any effort above the minimum level, which, if anticipated by the employers, leads to minimal wages. However, both employer and employee are better off if the employer trusts the employee by offering a wage above the minimum and the employee pays back that trust by exerting a higher effort. The *Lending Game* (Camerer and Weigelt, 1998) studies reputation formation in an incomplete-information setting where a borrower (whose type is unknown) interacts with several lenders, but each bilateral interaction displays the elements of a trust situation. Among all these and other games, however, it is Berg et al.'s (1995) game, and the label *Trust Game*, which has established itself as the most prominent instrument to measure trust in the laboratory, resulting in a large number of experimental replications and variations (see, e.g., Glaeser et al., 2000).

We would like to emphasize that the Trust Game and all the variants mentioned above arose from the discipline of *game theory*, and hence it might be worth providing some additional context at this point. This extensive, highly-developed, interdisciplinary field covers the formal and empirical study of interpersonal, strategic relations among multiple agents (see, e.g., Fudenberg and Tirole, 1991). For instance, *normal form games* are strategic situations where all involved agents act simultaneously. Starting with binary-action games (where a number of players have just two different actions each, the simplest case being two-player, 2 ×2 games), their study allows research in many relevant social issues, as coordination in efficient technologies. A case of particular interest is the study of cooperation in society using famous paradigms as the *Prisoner's Dilemma* (see Poundstone, 1993, for a detailed overview) or public good games (e.g., Fehr and Gächter, 2000). Indeed, large parts of the literature in social sciences beyond economics has often focused on 2 ×2 games as the Prisoner's Dilemma (e.g., Axelrod, 1984, 1997).

The Trust Game, however, is an *extensive form game*. This class of games allows to incorporate non-simultaneous play, and in particular reactions to previous actions of other agents (see Alós-Ferrer and Ritzberger, 2016, for a detailed formal treatment). The simplest examples, where all actions are observable, include paradigms which have been intensively used in *behavioral game theory* (see, e.g., Camerer, 2003) to investigate prosocial behavior, i.e., deviations from selfishness. For instance, in the Ultimatum Game (Güth et al., 1982) a proposer can offer a split of an endowment among two players, and a responder can then either accept it or destroy the entire endowment. On the basis of purely monetary payoffs, the normative *subgame-perfect equilibrium* predicts that the responder will accept any positive amount and, anticipating this, the proposer will offer as little as physically possible. In contrast, laboratory experiments show that human proposers make substantial offers and human responders reject small but positive offers. This does not, however, constitute a demonstration of prosocial behavior, since proposers might make positive offers strategically, to avoid rejections. For this reason, in the Dictator Game (Forsythe et al., 1994), which we will refer to in the following sections, the responder is passive and the proposer's decision is dictatorially implemented. Still, in this game human dictators typically grant positive amounts to the other player, in a striking deviation from selfishness.

*Evolutionary game theory* (see, e.g., Weibull, 1995), has focused on stylized games played in populations of agents to study the long-run evolution of fundamental features of society. Those include the evolution of cooperation (Nowak and Sigmund, 1993) and social preferences (Binmore et al., 1995; Miyaji et al., 2013), but the study of trust is so far underrepresented in this field. Although this subdiscipline has developed in economics and mathematical biology, it has recently been the subject of increased attention in other disciplines (e.g., Tanimoto, 2015, 2019).

In this review, we examine the difficulties and confounds inherent in the Trust Game, which include social preferences (section 2), attitudes to interpersonal risk (section 3), and other factors leading to a lack of stability of the paradigm (section 4). We also emphasize the differences in (measurements of) trust and trustworthiness (section 5), and conclude by exploring the relations to alternative instruments, in the form of survey questions (section 6), neurochemical manipulations and neuroimaging (section 7).

## 2. Social Preferences as a Confound

In spite of its widespread use to measure trust and trustworthiness in the lab, the Trust Game is not exempt of critiques. An important one is the possible presence of motivational confounds, very especially in the form of other-regarding preferences (e.g., Fehr et al., 1993; Fehr and Schmidt, 1999). If a trustor is selfish, the decision to trust should be motivated exclusively by the belief that the trustee will reciprocate. However, since the amount transferred is magnified by a factor *K* > 1, an altruistic trustor might decide to transfer resources even if he or she does not expect any transfer back, since what the trustor sacrifices is far less than what the trustee receives. Additionally, since in Berg et al.'s (1995) game efficiency does not require that trust is repaid, even an efficiency-motivated trustor (Bolton and Ockenfels, 2000; Charness and Rabin, 2002) might be willing to make a transfer even when not expecting a return. A similar critique applies to the trustee's transfer as a measure of trustworthiness. It is unclear whether a trustee's decision to transfer back arises exclusively from the desire to reciprocate (which is what is usually understood as trustworthiness) or from unconditional other-regarding preferences (e.g., inequity aversion).

To address these possible confounds, Cox (2004) conducted a between-subjects experiment, with one of the treatments being a standard Trust Game (*K* = 3). Confounds in trustor motivation were addressed through a second treatment implementing a Dictator Game (Forsythe et al., 1994), where “trustors” make the same decision as in the Trust Game (in particular, the amount sent is tripled) but “trustees” are passive and cannot reciprocate. A majority of trustors in the Dictator treatment made positive transfers, but transfers were significantly larger in the Trust treatment (on average, $5.97 vs. $3.63). Confounds in trustee motivation were addressed through a third treatment where trustors were passive, receiving an endowment equivalent to the fraction kept by trustors in the Trust treatment (crucially, not framed as a transfer). Trustees received three times the remaining fraction (plus a fixed endowment) and could send an amount to the passive trustors as in a Dictator Game. Roughly one third of the “trustees” in this treatment sent a transfer to the other player, even though reciprocity was not a factor. However, trustee average transfers were significantly larger in the Trust treatment ($4.94 vs. $2.06). This suggests that trust and reciprocity are indeed present as a motivation in the trustors' and trustees' decisions, respectively, but they are not isolated by the paradigm. On the contrary, a non-negligible part of the transfers of both types of agents might be motivated by prosocial preferences. For the case of trustors, this was confirmed by Chaudhuri and Gangadharan (2007), who ran an experiment including a Trust Game and a Dictator Game (see section 5 below). Again, transfers were significantly larger in the Trust Game (on average, $4.33 vs. $1.345). Further, the difference between the amount sent in the Trust Game and the amount sent in the Dictator Game was predicted by the elicited expectation for a back transfer from the receiver.

In conclusion, it might be unwarranted to use behavior in the Trust Game as a pure indicator of trust or trustworthiness. Due to the confound with social preferences, this game might be overestimating both dimensions of human behavior. To remove the confound, one should rely not on transfers in the Trust Game, but (whenever possible) on the within-subjects differences between those transfers and transfers in control games inspired on the Dictator Game. This might, of course, create difficulties of its own. For instance, Ashraf et al. (2006) report order effects depending on whether players played the Trust Game or a Dictator Game first.

## 3. Trust and Interpersonal Risk

Trusting someone puts you in a vulnerable position. By definition, the decision to trust implies assuming a risk. Hence, it is natural to ask whether attitudes toward risk influence the willingness to trust and hence whether there is another confound when measuring trust through the Trust Game. For instance, Houser et al. (2010) investigated the relation between trust and risk, measuring attitudes toward risk through the standard procedure of Holt and Laury (2002). However, the study did not find any systematic relation between trust decisions and risk attitudes. In contrast, risk attitudes did explain behavior in “risk games” where the trustee's decision was replaced by a known distribution. In a related study, Fairley et al. (2016) used a *risky Trust Game* as follows. Trustees' binary decisions to either keep the transfer or return half (independently of which proportion of the endowment was transferred) were elicited in advance. Trustors were told they would be matched with one out of four pre-determined trustees, and asked to provide their decisions conditional on how many of those trustees had decided to make a reciprocal transfer (Conditional Information Lottery design; Bardsley, 2000); hence, they provided five separate decisions. In practice, the trustor's decision was equivalent to a lottery. Behavior was compared to that in a standard Trust Game with no information on the trustee. Behavior in the risky Trust Game was used to estimate risk attitudes, and the resulting values do predict behavior in the Trust Game, although a standard measure obtained using Holt and Laury's (2002) procedure did not.

The lack of relation between risk attitudes and behavior in the Trust Game might be due to the fact that the risk involved in the Trust Game is of two qualitatively different kinds. On the one hand, there is the purely financial, dispassionate one, i.e., the risk to lose the money invested. On the other hand, there is a more psychological but not less-real risk, namely the risk to be betrayed by the trustee. More generally, attitudes toward risk in social and non-social situations might differ. To investigate this question, a number of studies have compared behavior in the Trust Game with behavior in risky situations where the social component is eliminated, but are otherwise equivalent to the Trust Game (in terms of outcomes) from the purely individual point of view.

Bohnet and Zeckhauser (2004) coined the term “betrayal aversion” to refer to the social aspect of risk in the Trust Game. In their study, they examined the question of whether the decision to trust a stranger is equivalent to taking a risky bet, or, on the contrary, the possibility of being betrayed by another human being represents an actual cost. For this purpose, they considered a mini-Trust Game as in Figure 1D, with *S* = *T* = 10, *B* = 8, *C* = 22, and *G* = *H* = 15 (and an implied *K* = 3). A distribution of responses (conditional on being trusted) was previously elicited from a population of trustees, resulting in an actual proportion of trustworthy players, *p* (unknown by trustors). Then, instead of a binary decision, a Minimum Acceptable Probability (MAP) was elicited from each trustor, with the explicit meaning that the trustor would actually trust if and only if the expressed MAP was larger than *p*. That is, if the MAP was larger than *p*, the trustor's decision would be implemented as “trust,” and the game's payoffs would be (*G, H*) with probability *p* or (*B, C*) with probability 1−*p*. If the MAP was smaller than or equal to *p*, the trustor's decision would be implemented as “mistrust.” The actual implementation varied across three variants of the game. In the actual Trust Game, the implementation was done by actually matching the trustor with a random trustee from the distribution, so that the outcome depended on the actual decision of the selected trustee. In a risky Decision Problem (framed as such), the outcome was implemented through a lottery with probabilities (*p*, 1−*p*), and, independently of the outcome, nobody received the trustee's payoffs; that is, in this case the participants chose between the safe payoff *S* and a lottery paying *G* with probability *p* and *B* with probability 1−*p*. The third variant was a Dictator Game which was identical to the risky Decision Problem, with the only difference that the trustee's payoffs corresponding to the actual outcome were received by an uninvolved, passive player.

The results of Bohnet and Zeckhauser (2004) showed a larger MAP for trusting in the Trust Game than for taking the risky option in the other games (but there were no differences among the latter two). Hence, participants revealed an aversion to experience betrayal in the Trust Game, separate from the non-social component of risk attitudes. This is an important result for the understanding of what the trustor decision actually measures in the Trust Game. Together with the results discussed in section 2, the results of Bohnet and Zeckhauser (2004) indicate that the decision to trust can be decomposed into a purely prosocial motivation and the willingness to assume risks of an interpersonal, social nature.

The relevance of betrayal aversion has been established in other studies. For instance, in a study using functional Magnetic Resonance Imaging (fMRI), Aimone et al. (2014) studied trusting behavior while controlling for social preferences. In their study, trustors played binary mini-trust games for 41 trials against human trustees whose decisions had been previously elicited, and for 41 further trials against random computer-generated decisions. Crucially, however, in the latter case another human being actually received the corresponding trustee payoff. Hence, the within-subject comparison controls for social preferences, but the trials with a computerized opponent should remove betrayal aversion. In line with Bohnet and Zeckhauser (2004), trust was observed significantly more often when playing against the computer (63%), compared to the trials with a human opponent (49%), although the effect was driven by male trustors.

Evidence to the contrary was presented by Fetchenhauer and Dunning (2012), who confronted participants with a binary-choice mini-Trust Game (as in Figure 1D with *S* = 5, *T* = 0, *B* = 0, *C* = 20, and *G* = *H* = 10, with an implied *K* = 4) and the choice to play an equivalent lottery. Trustee decisions (conditional on trust being shown) were collected in advance, and two different pools of decisions were created, containing 80 and 46% of trustworthy answers, respectively (High Chance and Low Chance conditions). Trustors were informed that the trustee's answer to their own decision would be extracted from the corresponding pool. Trustors also made an equivalent lottery choice decision, namely either to receive *S* = *$*5 for sure or a lottery paying *G* = *$*10 with either 80 or 46%, and zero otherwise. While there were no significant differences in the High Chance condition, in the Low Chance condition there were large differences (28.6% gambling in the lottery vs. 54.3% risking to trust in the Trust Game). That is, when the chances of winning were moderate, the decision to take the risk was made more often if the risk had a social component.

The results of Fetchenhauer and Dunning (2012) are in striking contrast to those of Bohnet and Zeckhauser (2004). The latter found that trust was increased when betrayal by a human being was not possible, i.e., there was less trust (as implied by a higher MAP) in the Trust Game compared with the risky version. Fetchenhauer and Dunning (2012) found that trust was reduced in the risky version of the game compared to the Trust Game, as revealed by the binary decisions on whether to trust or not. Those authors argue that the difference accrues from the elicitation methods. The MAP might elicit (abstract) betrayal aversion, but people are reluctant to openly signal distrust within the actual game. In light of these findings, further research should concentrate on clarifying the effects of betrayal aversion under different elicitation methods. There are, however, other differences in the designs which prevent a direct comparison. As also pointed out by the authors, in the design of Fetchenhauer and Dunning (2012) a trustor's mistrust decision gives the trustee zero payoffs and yields an unequal outcome (*S* = 5, *T* = 0), while in Bohnet and Zeckhauser (2004) in this case both players receive the same, positive payoff (*S* = *T* = 10). Hence, trustors might simply be reluctant to take the responsibility to leave the trustee empty-handed. A more complex argument would point out that the decision to trust in Fetchenhauer and Dunning (2012) “saves” the trustee from zero payoffs and might elicit stronger reciprocity motives than in Bohnet and Zeckhauser (2004), which in turn might be anticipated by the trustors. This is, however, at odds with the fact that trustors knew both that trustees' conditional decisions had been collected in advance *and* the percentage of trustworthy answers. We remark also that the sign of the difference found by Fetchenhauer and Dunning (2012) is consistent with the social-preferences confound described in section 2: since there were no trustees in the risky version, one could speculate that the higher transfers in the Trust Game of Fetchenhauer and Dunning (2012) might have been due to altruistic motivations which would naturally be absent in the risky version, where no trustee was present. However, this is inconsistent with the fact that there were no differences in High Chance condition.

In conclusion, betrayal aversion might be one of the main motivations behind the decision (not) to trust. Since this reflects a particular kind of *social* risk, researchers should be aware that standard measures of risk attitudes might not be well-suited to the study of trust. At the same time, this observation shows an essential defining characteristic of experimental paradigms measuring trust, in the sense that potential game “variants” which remove or weaken the social aspect of the trusting decision might very well end up measuring unrelated characteristics.

## 4. Lack of Stability of the Paradigm

In this section, we discuss current evidence showing that minor changes in the parameters, implementation, and description of the Trust Game might sometimes induce large changes in players' responses. This is problematic, as it suggests that the paradigm might not be as stable as would be desirable for an instrument measuring an aspect of human motivation.

A first example is the size of the multiplier, which was set to *K* = 3 in the original version of Berg et al. (1995). Lenton and Mosley (2011) found evidence that increasing the multiplier (from 2 to 3 or 4) increases the fraction of the endowment sent by the trustor. Some studies have shown that increases in the multiplier also increase the fraction returned by the trustee (reciprocity), comparing e.g., 3 vs. 6 (Ackert et al., 2011) and 2 vs. 4 (Mislin et al., 2015). However, the meta-analysis of Johnson and Mislin (2011), which included trust games with many implementation variations, reached the puzzling conclusion that increasing the multiplier from 2 to 3 *decreases* the trustee's transfers, but it does not affect the trustor's transfers. This finding coexists with the observation that trustees respond to larger fractions transferred by the trustors by transferring back larger fractions of the income they receive.

A second factor which might affect behavior in the Trust Game, specifically trustees' transfers, is whether answers are elicited through the strategy method (Selten, 1967) or as answers to the actual trustor decision. In the former, trustees are asked what their return transfer would be conditional on each possible transfer of the trustor (before the actual one is revealed), and trustor-trustee decisions are paired afterwards. In the latter, trustees are confronted with the actual trustor decision and asked to react to it (and only to it). It has been argued that the strategy method might in general induce more deliberative, “cold” thinking in experiments (Brandts and Charness, 2000), and in particular Casari and Cason (2009), argue that this method might reduce transfers of trustees in the Trust Game. However, Brandts and Charness (2011) found no difference.

Another example is framing. Burnham et al. (2000) consider an extensive form game where each player has multiple decisions but the first two decisions roughly correspond to a trust situation. They show that players in the role corresponding to a trustor trusted more if the instructions called the other players “partners” rather than “opponents.” However, in many implementations of the Trust Game, the word “Trust” is not mentioned at all, hereby avoiding framing effects. In an EEG experiment, Sun et al. (2019) framed a (repeated) Trust Game either literally as a “Trust Game,” or alternatively as a “Power Game,” and found that earnings (and hence trusting behavior) were larger in the first case.

A subtler issue related to framing is that the way the instructions of a game are spelled out might influence how participants interpret the situation, and also whether a shared interpretation arises. As pointed out by Ermisch and Gambetta (2006), even the attempt to keep the game frame-free raises the concern that trustees might develop other, alternative interpretations. A demonstration of the effects of a shared interpretation was given by Cronk (2007), who conducted trust games among Maasai natives, with *K* = 3 and the initial trustor endowment *X* corresponding to about a day's wage at the time. Half the games were kept unframed, and the rest were explicitly called *osotua* games. This word describes a strong, culture-specific concept where a request of a gift or favor arises out of genuine need, and granting it creates a strong, long-lasting bond. Both trustor's transfers and trustee's returns were smaller in the *osotua* treatment. Crucially, there was a *negative* correlation between the trustor's transfer and the trustee's return share, which was absent in the unframed treatment. This is natural given that *osotua* refers to freely-given gifts in case of genuine need, but in the absence of this culture-specific information, the evidence might be misinterpreted as reduced trust and trustworthiness.

Chaudhuri et al. (2016) conducted several laboratory treatments using trust games (*X* = 10, *K* = 3) with incremental differences in the instructions. Some of the treatments provided context by explicitly spelling out the conflict underlying the trustor's decision. Specifically, the instructions contained two paragraphs which described the subgame-perfect outcome (where the trustor “sends” zero) and the fact that both players would be better off if the trustor sent the entire endowment of 10 units and the trustee returned more than 10 units back. The trustors' transfers were significantly larger in the context-rich treatments, compared to a control. Importantly, also the trustees' return shares were larger in the context-rich treatments, i.e., trust did pay off. At the same time, there were no significant differences across the context-rich treatments even though two of them, but not the third, included the words “trust” and “trustworthy.” That is, the framing effect was not due to the use of particular words but rather to the fact that the conflict between self-interest and the maximization of social surplus was made evident. At a conceptual level, this study and Cronk (2007) suggest that players in the Trust Game (and, more generally, in many experimental paradigms) might often face some uncertainty on whether all involved participants share a common view of the game. Both cultural labels (shared by all participants) and explicit information on the nature of the conflict underlying player decisions help reduce such uncertainty.

In conclusion, mixed evidence on several fronts has cast doubt on the stability of the measures derived from the Trust Game. Further, systematic research is needed to clarify to what extent those measures reflect stable personality traits or rather situation-specific reactions. Comparability to the literature can only be guaranteed by relying on designs as close as possible to those used in the relevant, previous contributions. The issue of framing is particularly worrying, and simply striving to keep a neutral framing might not always be enough to ensure that the subjects' interpretation of the game coincides with that of the experimenter.

## 5. Trust vs. Trustworthiness

Trust and trustworthiness go hand-in-glove, as one cannot exist without the other. However, no matter how interrelated they might be, they are clearly different concepts. For instance, while monetary concerns might partially explain the decision to trust (accepting interpersonal risk in order to obtain a higher return), they cannot explain the decision to repay trust, as the selfish, profit-maximizing decision is always to keep the entire transfer received. Hence, one should expect that trust and trustworthiness are explained by partially different determinants. A key contribution on this front is Ashraf et al. (2006), who confronted *N* = 359 college students from different countries with a Trust Game, two dictator games, and a number of questionnaires (including measurement of risk attitudes). Half of the participants played the Trust Game (*K* = 3) as trustors, and the rest as trustees. Trustors were also asked what they expected to get back, as a measure of their expectation of trustworthiness. The dictator games were a regular one and a “Triple Dictator Game” identical to the Trust Game (so the amount sent by the first player was tripled) except that the second player was passive and could not return anything back (as in the Dictator treatment of Cox, 2004). The amount sent in the Triple Dictator Game is used as a proxy of “unconditional kindness” or prosocial behavior for the trustors, while the amount sent in the regular Dictator Game plays the same role for the trustees.

The first observation is that, out of the 159 trustors who sent a positive amount, only 36% expected back more than what they sent. This suggests that most people exhibit trusting behavior due to motivations other than purely monetary ones. As in Cox (2004) (recall section 2), trustor behavior is partially explained by prosocial behavior (the amount sent in the Triple Dictator Game). However, a regression analysis shows that most of the variance in trustor transfers is actually explained by the expectations of trustworthiness. On the other hand, trustees' return transfers are explained both by trust shown (the amount sent by trustors) and prosocial behavior (the amount sent in the regular Dictator Game). The latter is a restatement of the observation that, as trust, trustworthiness as measured in the Trust Game is confounded with prosocial behavior (recall again section 2). As for the former, the relation between the trustor's transfer and the trustee's return (elicited here through the strategy method) is commonly taken as a demonstration of reciprocity. Ashraf et al. (2006) further argue that trustees' transfers are better explained by prosocial motivation than by reciprocity, because, in a regression analysis, the amount sent in the Dictator Game explains most of variance in trustees' transfers, compared to a model where the trustor's transfer is also included as a regressor. Interestingly, the authors also consider a different measure of other-regarding preferences, namely the “predicted distributional preference.” This is the amount that the trustee would need to return to the trustor to create the same payoff ratio as the trustee created in the regular Dictator Game. Hence, it is a function of both the trustor's transfer and the amount sent by the trustee in the Dictator Game. When included in a regression, this variable captures almost the same variance as that explained in a model including both of the latter variables.

Since the determinants of trust and trustworthiness are different, it is natural to ask whether, at the individual level, being more trusting implies that one is also more trustworthy, and vice versa. The Trust Game measures trust and trustworthiness as the behavior of the two different players, trustor and trustee. Hence, the relation between trust and trustworthiness at the individual level can be tackled by relying on experimental designs where participants play both roles (in different trust games).

Chaudhuri et al. (2003) let 76 participants play both roles in a bargaining game with a structure akin to a mini-Trust Game, but with the option for the trustee to (costly) punish if no trust was shown (an option that nobody used). Of the 39 participants who trusted their counterparts and were themselves shown trust when in the trustee role, 18 did not reciprocate, suggesting that people who trust are not necessarily trustworthy. This hypothesis was confirmed in Chaudhuri and Gangadharan (2007), who collected data from participants who played both roles in trust games (*X* = *$*10, *K* = 3). Dividing participants into trusting and non-trusting depending on whether they transferred 50% or more (*N* = 42) or less than 50% (*N* = 58), respectively, they found no significant differences in their average return transfers (16 and 18%, respectively). On the other hand, dividing participants into trustworthy and less trustworthy depending on whether they returned one third or more (*N* = 27) or less than one third (*N* = 55) of the amount actually offered to them (*N* = 18 received zero), they found that the trustworthy participants sent significantly more as trustors than the less trustworthy ones ($5.33 vs. $3.82 on average). That is, while trustworthy participants were found to be generally trusting, there was no evidence that more trusting individuals are also necessarily more trustworthy.

Chaudhuri and Gangadharan (2007) argue that what has been interpreted as trust in many studies could be decomposed in two components. One essentially corresponds to the predisposition to accept a social risk that we discussed in section 3, and that obviously plays a role for trustors but not trustees in the Trust Game. The other is a general prosocial orientation, related to the social-preferences confound that we discuss in section 2, and that could be considered a “social virtue” in the sense of Fukuyama (1995). For instance, participants in the experiment of Chaudhuri and Gangadharan (2007) also played as dictators in a Dictator Game, and trustworthy participants transferred significantly more in the latter game than less trustworthy ones ($1.89 vs. $0.83, respectively). Chaudhuri and Gangadharan (2007) conclude that trustworthiness as measured in the Trust Game might be more relevant than trust for the study of social capital and its relation to economic growth.

In conclusion, trust and trustworthiness are interrelated but different concepts, influenced by different individual characteristics and factors. There is evidence suggesting that trustworthy individuals might be generally more trusting, but the converse is in general not true.

## 6. Surveys and the Trust Game

Besides experimental paradigms as the Trust Game, the most common method for measuring trust is the use of generalized trust questions in surveys. The most prominent example is the General Social Survey (GSS) of the U.S. National Opinion Research Center (http://gss.norc.org), which has collected evidence on trust and social capital since 1972. The specific question used to measure trust was adapted from Rosenberg's (1956) misanthropy index (see Uslaner, 2012) and reads as follows. “*Generally speaking, would you say that most people can be trusted or that you can't be too careful in dealing with people?*” The question is a binary-choice one with two possible answers, “*Most people can be trusted*” or “*Can't be too careful*” (plus “I don't know”). This question is used in many other surveys, as, e.g., the European Values Survey, the World Values Surveys (WVS), the British Household Panel Study (BHPS), and American National Election Studies (ANES).

The question, however, is not exempt of criticism. Obviously, responses to those depend on the respondents' interpretation of the questions and their personal experiences. The question has been criticized for being too generic (Ermisch et al., 2009) and for reducing a presumably-continuous characteristic to a dichotomous answer (Lundmark et al., 2015), although Uslaner (2012) points out that binary answers avoid “clumping” in intermediate options. Most importantly, Miller and Mitamura (2003) have pointed out that the two alternative answers refer to an assessment of other people's trustworthiness and an assessment of one's own willingness to take risks, respectively. That is, respondents are forced to choose between trust and caution, although these options are not opposed (recall section 3).

Glaeser et al. (2000) tested whether attitudinal trust questions from surveys predict actual, incentivized trusting behavior in the Trust Game. Maybe unsurprisingly, there was no relation between the answers in survey questions and trustor's behavior. There was, however, a correlation between the answer to the GSS question and trustworthiness as measured by trustees' behavior in the Trust Game. Trustor behavior correlated with answers to different questions on placing trust on strangers. Similar results have been obtained by Ashraf et al. (2006), Lazzarini et al. (2005), and Ermisch et al. (2009) (using a representative sample from the BHPS). In contrast, the representative-sample studies of Fehr et al. (2003) (using the German Socio-Economic Panel, SOEP) and Bellemare and Kröger (2007) (using the Dutch CentERpanel) found that survey questions about people's trust and especially past trusting behavior are predictors of trusting behavior. In summary, evidence is mixed, and the relation between the two measures of trust is unclear at this point. A possible preliminary conclusion in view of the evidence is that the Trust Game tests a very specific, strategic situation and trustor behavior might not be a good indicator of the generalized form of trust captured by the GSS question or related ones.

Since our understanding of the relation of various survey measures to behavioral measures of trust is still limited, it might be worth exploring survey questions in populations comparable to those of standard laboratory experiments. For instance, Chaudhuri et al. (2016) replicated one of the context-rich treatments of their experiment (discussed in section 4) and found a significant positive correlation between trustors' transfers and their responses to a five-item questionnaire by Yamagishi (1986), but the questionnaire was not related to the decision to reciprocate. Ben-Ner and Halldorsson (2010) examined both survey measures and behavior in the Trust Game with a sample of university students (*N* = 204). They found a weak but significant correlation between the amount sent by trustors in the Trust Game and the answer to the GSS question. By examining the impact of a battery of measures (including both factors determined at birth and factors determined by attitudes, views, and social preferences) on behavioral and survey measures of trust and trustworthiness, they suggest that trustor behavior in the Trust Game and survey measures of trust might capture different facets of a richer (but unspecified) construct. Trustee behavior might capture trustworthiness for investment situations, but certain survey measures, for instance the Machiavellian scale (Christie and Geis, 1970), appear to reflect different facets of trustworthiness.

Recently, Falk et al. (2018) conducted the *Global Preference Survey (GPS)* eliciting a variety of preference reports from 80,000 people across 76 countries. Trust was measured through one self-report item asking respondents simply whether they assumed that other people have only the best intentions (Likert scale, 0–10). The scale correlated significantly at 0.49 with the GSS question, which could be interpreted as positive evidence on the validity of the latter. The main message, however, is that, as for many other indicators of individual preferences, there is a large heterogeneity in trust, with variation arising from both individual and aggregate characteristics (cultural and geographical). Regarding individual characteristics, unsurprisingly, trust as measured in the GPS correlates with measures of altruism and positive reciprocity, but it also correlates positively with measures of patience and negative reciprocity. In almost all countries, trust increased with cognitive ability. At the purely demographic level, older individuals tended to be more trusting as measured by the GPS, but gender effects where less clear, with differences (in favor of women being more trusting) being significant in only one third of all countries. Interestingly, trust increased in the presence of domestic animals. Concerning aggregate characteristics, trust correlated with latitude, with trust levels being particularly high in the USA, Canada, and Australia. Last, trust was a positive predictor of economic development (as proxied by income per capita), in a country-level regression, but the relation became non-significant when controlling for patience.

In view of the country differences found by Falk et al. (2018), it is interesting to recall the results of Yamagishi and Yamagishi (1994), which called into question previous findings showing higher levels of trust, as measured by survey scales, in the USA than in Japan. This is in contrast with the conventional image of Japanese society, where mutual trust and stable long-term relationships (both social and economic) are highly appreciated. In stark contrast to single-item measures, Yamagishi and Yamagishi (1994) used an extensive (86-item) questionnaire in both countries to examine differences in the concept of trust. They proposed to distinguish a concept of *general trust* from a more specific concept of *assurance*. The former refers to the evaluation of potential partners in the presence of incomplete information and social uncertainty. The latter refers to a need for the reduction of social uncertainty, mostly through the formation of mutual-commitment relations (which might lead to foregoing new opportunities). While Americans scored higher than Japanese in general trust, the opposite was true for assurance. That is, Japanese place a higher value on the long-term aspect of trust, which emphasizes forming lasting, stable relationships. This study serves as a word of caution on cultural differences in the concept of trust.

In conclusion, survey measures appear to be only weakly related (if at all) to behavior in the Trust Game. A possible interpretation of the state of the literature is simply that the various behavioral and survey measures capture different facets of generalized, abstract notions of trust and trustworthiness. Hence, researchers should not assume that any particular behavioral or survey measure available at this point suffices to cover all aspects of our intuitive notions of human trust and trustworthiness.

## 7. The Neuroscience of Trust

As illustrated in the previous sections, behavioral (choice) studies have made abundantly clear that trust is a multifaceted concept which interacts with many other aspects of social behavior (which are potential confounds). As a consequence, behavioral and self-report measures might be too simple to capture trust at the individual level in a stable and reliable way. It is natural to ask whether more objective, biological correlates exist. Recent advances in neuroscience point at two natural avenues of research. On the one hand, the hormone oxytocin has been shown to be related to trusting behavior. On the other hand, brain scanning studies are shedding light on the neural correlates of trust.

### 7.1. Oxytocin and Trust

The neuropeptide hormone oxytocin (OT), synthesized in the hypothalamus, is known to modulate social behavior both in humans (IsHak et al., 2011) and non-human animals (Donaldson and Young, 2008), enabling pair bonding (Young and Wang, 2004) and maternal attachment (Insel and Young, 2001). Indeed, OT has been popularly labeled as “love hormone” or “liquid trust” (Nave et al., 2015).

The literature can be usefully divided into correlational studies, which exploit the endogenous variation in OT levels in blood (Zak et al., 2005), saliva (Tops et al., 2013), or urine (Ebert et al., 2013)^1^ and causal studies, which produce exogenous variation by administering OT, via either intranasal (Kosfeld et al., 2005; Baumgartner et al., 2008) or intravenous routes (Hollander et al., 2007; Lee et al., 2018).

Both approaches have been used to link OT levels and behavior in the Trust Game. Zak et al. (2005) was the pioneering correlational study, measuring natural variation of OT levels in blood samples immediately after subject decisions in a Trust Game with *X* = *$*10 and *K* = 3 (as in Berg et al., 1995). This was compared to a Random Draw condition where the trustor's transfer was determined as a random integer from 0 to 10. Trustee's OT levels in the Trust Game were 41% higher than in the Random Draw condition. In the Trust Game, the amount sent back by the trustees was a function of the amount sent by the trustor and the log of OT levels (the logarithm form is expected to capture saturation). However, log OT was not statistically significant in the Random Draw condition. Hence, OT levels can be seen as a correlate of reciprocity (trustworthiness), in the sense that they correlate with return transfers but only if those respond to an actual, intentional transfer. However, OT levels did not predict trustors' transfers, that is, the evidence of Zak et al. (2005) refers exclusively to trustee behavior. The basic message of this and other studies is that being treated well (e.g., shown trust) results in OT production, which in turn increases reciprocity. This has motivated a recent “neuromanagement” view (Zak, 2017, 2018) which tries to spell out the possible changes in organizational culture which can (presumably through induced oxytocin release) promote trust and prosocial behavior within the organization.

Studies exploiting endogenous OT variation, though, cannot establish causality. Kosfeld et al. (2005) was the first causal study testing the hypothesis that OT increases trusting behavior in humans using the Trust Game. A single dose of either OT or a placebo was administered intranasally to each study participant. Then they played a Trust Game with *X* = 12 monetary units and *K* = 3, with trustor transfers constrained to being multiples of four (trustee transfers were unconstrained; additionally, trustees had a supplementary endowment of 12 units). The results showed significantly larger trustor transfers for participants who received an OT dose, compared to those who received placebo. In contrast, there were no significant differences in the levels of reciprocity (return transfers from the trustees) between the OT and the placebo group.

Further, the Trust Game in Kosfeld et al. (2005) was contrasted with a later Risk experiment, where the transfer was framed as an individual, risky investment and the returns to the investors were determined by a random device (there was no second player). Specifically, the random device reproduced the distribution of decisions from the trustees in the previous Trust Game experiment, conditional on each trustor transfer level (recall section 3). In this game behavior did not differ between OT participants and placebo ones. Hence, the results suggest that OT causally increased trust (and not simply risk-taking behavior). However, since there was no second player in the risk experiment, one may ask whether OT simply increases prosociality in general. This appears unlikely, since trustees' behavior was unaffected by OT administration. This suggests that OT administration differentially influences trust, but not reciprocity or general prosocial behavior. Further, trustors were asked about their beliefs on the trustee's transfers, and again the OT group and the placebo one did not differ. Thus, OT did not alter trustor's beliefs (e.g., making them more optimistic). Hence, the natural conclusion is that the mechanism by which OT administration increases trusting behavior is a reduction of betrayal aversion (recall section 3).

The latter conclusion was strengthened by the work of Baumgartner et al. (2008) (see also Fehr, 2009), who administered OT or placebo intranasally to participants who took the trustor role in the Trust Game while undergoing functional magnetic resonance imaging (fMRI). The Trust Game was implemented as in Kosfeld et al. (2005), with the only difference that trustees had just the binary option to either betray the trustor by keeping the whole transfer or honor trust by making a return transfer which equalized payoffs (this was made possible by the trustee's endowment of 12 units). The experiment also included a Risk Game with equivalent binary return transfers. In both conditions, return transfers occurred 50% of the time (ensured by using previously-elicited answers from trustees in a pilot experiment). The focus of Baumgartner et al. (2008), however, was on the reaction of trustors to feedback, implemented as follows. Trustors first played 6 Trust Games against different trustees and 6 risk games, in random order. Trustors were then informed that half of the time there had been no return transfers (with some randomness added if trust or investment happened an odd number of times). After this feedback, they again played 6 Trust Games and 6 risk games. In the post-feedback phase, trust levels (as measured by transfers) decreased in the placebo group, but *not* in the OT group. That is, experiencing previous betrayals did not reduce trust when OT had been administered.

As the discussion above illustrates, there is a fundamental inconsistency between the results arising from the two methods to study the relation between OT and trust. The study of Zak et al. (2005) finds that naturally-occurring levels of OT predict trustee behavior (reciprocity) but not trustor behavior (trust), venturing the explanation that being shown trust increases OT levels, and those lead to reciprocity. However, the latter link is absent in the data of Kosfeld et al. (2005), who found that OT administration increases trustors' transfers (trust) but does not influence trustee behavior. More recently, Nave et al. (2015) cast doubts on previous results. Pooling the data of seven different experiments where OT had been administered intranasally to participants in the Trust Game, the authors found no robust results in the aggregate. However, it is too early to draw conclusions. As cautioned by Nave et al. (2015), failed replications might be linked to technical difficulties with intranasal OT administration, and the effectiveness of the method itself is still not fully established because, at this point, it is not entirely clear how OT reaches the human brain after administration.

The relation between OT and trust or trustworthiness might simply be more complex than initially assumed. Zhong et al. (2012) measured blood OT levels in 1,158 Chinese undergraduates and found evidence for a non-monotonic (U-shaped) relationship. Subjects in the top *and* bottom 20% of the OT distribution made significantly larger transfers both as trustors and as trustees (respectively, 15.6 and 8.3%) than those in the middle 20% of the distribution. However, participants in the study played both roles, which could have led to spillovers. Also, the relation might be context-dependent. Mikolajczak et al. (2010) provided trustors with stereotypical descriptions of trustees which emphasized reliability or lack thereof. Intranasally-administered OT increased trust compared to a placebo group, but only when the trustee was described as reliable.

Other studies have explored the relation between OT and other dimensions of social behavior, beyond the specific concepts of trust and trustworthiness captured by the Trust Game. Theodoridou et al. (2009) find that OT administration increases judgments of trustworthiness and ratings of attractiveness of pictures of faces. Shamay-Tsoory et al. (2009) found that OT increased envy and *Schadenfreude* when observing own payoffs and the (purported) payoffs of another participant, which is, at least conceptually, in contrast with the results reviewed above. Domes et al. (2007) showed that OT improves the ability to infer the mental states of others from pictures of the eyes regions (“Reading the Mind in the Eye Test,” Baron-Cohen et al., 2001).

Recently, Marsh et al. (2017) asked German male students to make (costly) donations for either refugees (outgroup) or locals (ingroup) in need using (truthful) vignette-based descriptions. OT administration resulted in higher donations toward both groups, compared to placebo. Participants completed a version of the xenophobia scale of Schweitzer et al. (2005), and those scoring lower (according to a median split) more than doubled their contributions to both ingroup and outgroup under OT, but the peptide had no effect for those scoring higher in the xenophobia scale. Also, those scoring lower donated 31% more to the outgroup than to the ingroup (irrespective of the OT treatment), but again there was no difference for those scoring higher. Importantly, in a second donations round social norms were manipulated by (truthfully) reporting the average donations per vignette from a previous experiment where donations to the outgroup were 19% higher than those to the ingroup (this difference was achieved using reputation pressure). Strikingly, under OT, subjects who scored high in the xenophobia scale donated 74% more to the outgroup when the norm was manipulated, compared to the absence of a communicated norm. That is, neither the norm nor OT administration alone did influence outgroup donations for the high-scores group, but the conjunction of both manipulations was successful. This study effectively illustrates the main takeaways of the recent literature on OT. First, OT modulates a more general aspect of trust than the dimensions studied in the Trust Game. Second, it does so in a nuanced, context-dependent way which interacts with individual differences.

### 7.2. Neural Indicators of Trust

The decision to trust entails an evaluation of the expected actions that a different person will take in response. That is, one needs to anticipate the reactions of another decision maker, which requires the set of social-cognitive functions known as *Theory of Mind* (ToM; see, e.g., Singer and Tusche, 2014; Alós-Ferrer, 2018). The brain network underlying Theory of Mind is known to be built along a frontal-temporoparietal link, in particular including key areas as the medial prefrontal cortex (mPFC) and the temporoparietal junction (TPJ). Accordingly, the first fMRI study on the Trust Game, McCabe et al. (2001), targeted the mPFC. Participants played a series of binary-action games including mini-Trust Games, either with a human partner (outside the scanner) or against a computer that made stochastic choices following a given distribution. For the players who consistently made more “cooperative” (trusting) decisions, the study found increased mPFC activity when playing against human partners, compared to playing against the computer. In the Trust Game participants played both as trustor and as trustee (in different trials), but brain activity was analyzed in the time window corresponding to either the end of a decision period of a trustor or the end of the waiting period of a trustee (where presumably the participant was thinking about the trustor's action). Hence, it can be assumed that mPFC activity was linked to the decision whether to trust or not.

Krueger et al. (2007) pointed out that the role of the mPFC might be complemented by other, different brain regions depending on the strategies followed to establish trust in repeated interactions. Participants played a repeated, non-anonymous binary mini-Trust Game while alternating their roles as trustor and trustee. Consistently with McCabe et al. (2001) and the need for Theory of Mind, decisions to trust resulted in differential activation of the mPFC (paracingulate cortex), compared to decisions in a control “game” which involved no interpersonal interaction. Also, in alignment with the results discussed in the last subsection, the contrast also revealed differential activation of the septal area (and the adjacent hypothalamus), which contains oxytocin receptors and is involved in the releases of that peptide. Krueger et al. (2007) divided the experiment in two phases, assuming that the earlier and later one would correspond more to partnership building and maintenance, respectively. Participants were classified as defectors and non-defectors, depending on whether their groups experienced some or no defections during play, respectively. In the building-partnership stage (first-mover), non-defectors showed higher mPFC activation than defectors. However, mPFC activity decreased for non-defectors and increased for defectors over the course of the experiment. In the maintaining-partnership stage, non-defectors showed higher activation of the septal area than defectors, while the latter showed higher activation of the Ventral Tegmental Area (VTA) than the former. The VTA is part of the (dopaminergic) reward valuation network of the brain (see, e.g., Daw and Tobler, 2014). The interpretation is that defector and non-defector groups used different strategies. Defectors relied less on Theory of Mind in the building stage, resulting in comparatively lower levels of trust and lower payoffs. This negative reinforcement, through VTA involvement, resulted in later attempts to repair trust, adding up to a *conditional trust* strategy. Non-defectors, in contrast, employed an *unconditional trust* strategy which led to increased social attachment as reflected by activity in the septal area.

The role of the ToM network for trust is by now firmly established, to the point that the effect of various factors influencing trust might be better understood in terms of their effects on this network and the connectivity between its nodes and other brain areas. For example, Engelmann et al. (2019) showed that aversive affect, induced through prolonged periods of threat of shock, reduced trusting behavior in the Trust Game. The study also provides insights on the likely neural mechanisms underlying this result. Aversive affect reduced activity in the TPJ and also reduced functional connectivity between this area and the amygdala, which plays a key role in emotional processing.

It needs to be remarked that a further brain region, the anterior insula, may also play an important role for the decision to trust. This was shown by Aimone et al. (2014), who investigated the neural foundations of betrayal aversion (recall section 3). Participants in the trustor role showed significantly higher anterior insula activation when deciding to trust a human partner, compared to a computerized one, even though in the latter case the trustee payoffs were also received by a human being. In contrast, there was no difference when deciding not to trust. Hence, activity in the anterior insula might be crucial indicator of betrayal aversion. This is in agreement with data showing that the insula plays an important role in social emotions (Singer and Lamm, 2009).

The decision to reciprocate trust, as discussed in previous sections, presents major differences with the decision to trust. Consequently, it would not be justified to assume that the same neural processes underlie both decisions. An example of the specificities of reciprocity is given by King-Casas et al. (2005). In their study, a trustor and a trustee, with fixed roles, played a repeated Trust Game (*X* = 20, *K* = 3) consisting of ten consecutive rounds. Hence, even the trustor's decision involves a reciprocity component, as it can repay or betray the previous trustee decision. When playing in the trustee role, there was increased activity in the striatum (caudate head) when the trustor behaved generously (sending more in response to a previous trustee defection), compared to when the trustor defected (repaying the trustee's previous reciprocity with a decreased transfer). This might be reflecting a signal on the expectation of reciprocal behavior, consistent with current interpretations on the role of the reward prediction error for human decision making (Daw and Tobler, 2014). More generally, while trust might be motivated by the expectation of future reciprocity, reciprocal behavior will be influenced by the experience of trust, and specifically deviations from expectations.

However, the human decision to reciprocate clearly depends on the intentionality of the received transfer. The latter presupposes Theory of Mind. Hence, it would also be surprising to find no overlap between the neural substrates of trust and reciprocity. Van Den Bos et al. (2009) showed that key nodes of the ToM network also play an important role in reciprocity in the Trust Game. Specifically, reciprocity might reflect the interaction of anterior mPFC and the TPJ. In their study, participants played as trustees in a binary mini-Trust Game. Higher anterior mPFC activation was found when participants defected compared to when they reciprocated. In contrast, activity in the TPJ, bilateral insula, and anterior cingulate cortex (ACC) was modulated by individual differences in social preferences as captured by the Social Value Orientation incentivized scale (SVO; Murphy et al., 2011).

In addition to deepening our understanding of the processes underlying trust and reciprocity, recent research in neuroscience also might suggest a possible way of developing more reliable versions of individual heterogeneity in the underlying predispositions. Bellucci et al. (2019) show that (task-free) resting-state functional connectivity (RSFC) predict individual differences in both trust and reciprocity in a one-shot Trust Game. This is less surprising than it might seem at first glance, because the RSFC reflects the activity in the Default Mode Network (DMN), which displays a large overlap with the ToM network, including, e.g., the mPFC and the TPJ (see, e.g., Alós-Ferrer, 2018).

## 8. Conclusion

Trust and reciprocity are complex behavioral phenomena which interact with many other, different aspects of human social behavior. There might be multiple (but not necessarily mutually exclusive) definitions of trust, reflecting cultural, situational, individual, and neural differences. Quite possibly, there might even be disciplinary differences across the social sciences. The Trust Game is an ingenious but highly-stylized experimental paradigm, which has delivered important insights and remains an important benchmark. It is, however, too stylized to provide a complete picture of the nuances behind trust and reciprocity by itself.

The limitations of the Trust Game might be overcome by carefully controlling for known confounds, as prosocial motivations or social risk. Additionally, a number of complementary measures are readily available, even if none of them seems ready to become the new golden standard. Survey measures have their own problems, but are easy to administer and help acquire longitudinal data which are typically beyond reach when using laboratory-based methods. Neurochemical measurements (chiefly oxytocin) offer a different perspective which might open the door to causal interventions, although, in view of mixed results, caution should be advised at this point. Brain imaging studies allow us to identify direct, neural correlates with the potential to ultimately open the black box of why and how trust takes place.

In view of the literature, there is no doubt that the Trust Game will remain an important instrument in the social scientist's toolbox for many years to come. At the same time, that toolbox, and in particular the part used to measure trust and reciprocity, has grown significantly in the recent years, and it is not necessary to arbitrarily restrict attention to a particular instrument.

## Author Contributions

All authors listed have made a substantial, direct and intellectual contribution to the work, and approved it for publication.

### Conflict of Interest Statement

The authors declare that the research was conducted in the absence of any commercial or financial relationships that could be construed as a potential conflict of interest.

## Footnotes

^1^See McCullough et al. (2013) for a discussion of the accuracy of the different methods.
